# Neuropeptides: Roles and Activities as Metal Chelators in Neurodegenerative Diseases

**DOI:** 10.1021/acs.jpcb.0c11151

**Published:** 2021-02-11

**Authors:** Shira Ben-Shushan, Yifat Miller

**Affiliations:** † Department of Chemistry, Ben-Gurion University of the Negev, P.O. Box 653, Be’er Sheva 84105, Israel; ‡ Ilse Katz Institute for Nanoscale Science and Technology, Ben-Gurion University of the Negev, Be’er Sheva 84105, Israel

## Abstract

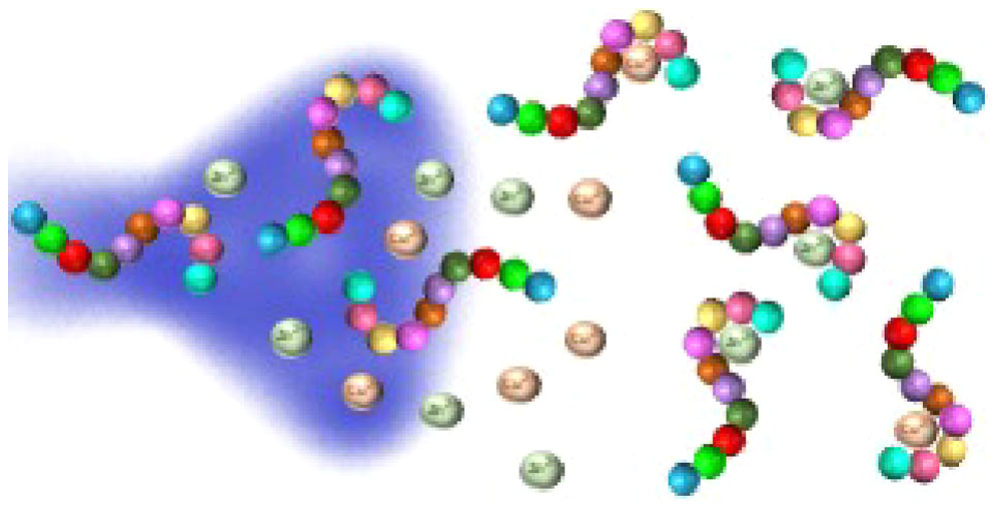

Neurodegenerative diseases, such as Alzheimer’s disease (AD) and Parkinson’s disease (PD), are characterized by deposits of amyloid proteins. The homeostasis of metal ions is crucial for the normal biological functions in the brain. However, in AD and PD, the imbalance of metal ions leads to formation of amyloid deposits. In the past four decades, there has been extensive effort to design compound agents than can chelate metal ions with the aim of preventing the formation of the amyloid deposits. Unfortunately, the compounds to date that were designed were not successful candidates to be used in clinical trials. Neuropeptides are small molecules that are produced and released by neurons. It has been shown that neuropeptides have neuroprotective effects in the brain and reduce the formation of amyloid deposits. This Review Article is focused on the function of neuropeptides as metal chelators. Experimental and computational studies demonstrated that neuropeptides could bind metal ions, such as Cu^2+^ and Zn^2+^. This Review Article provides perspectives and initiates future studies to investigate the role of neuropeptides as metal chelators in neurodegenerative diseases.

## 1 Introduction

The pathological self-assembly (or aggregation) of amyloid proteins into toxic aggregate species plays an important role in neurodegenerative diseases, e.g., Alzheimer’s disease (AD) and Parkinson’s disease (PD). Metal dyshomeostasis is well-recognized as a crucial factor in neurodegenerative diseases.^1−3^ The metals that play a role in the etiology of these diseases include divalent transition metals, such as Fe^2+^, Cu^2+^, and Zn^2+^ ions.

One of the hypotheses that has been proposed is that there are interactions between metal ions and amyloid, such as Aβ in AD and α-synuclein in PD.^4,5^ On the basis of this hypothesis, disruption of metal–amyloid interactions by metal chelation therapy has been proposed in order to reduce the neurotoxicity of metal–amyloid species and with the aim to restore metal homeostasis in the brain.^6^ However, in order to design metal chelators as potential drugs in the treatment of neurodegenerative diseases, the metal chelators must have appropriate characterizations. First, the chelators must have low molecular weight, uncharged molecules or have relatively poor charges to cross the blood–brain barrier (BBB) and be able to keep their stability. Second, metal chelators must selectively target specific metal ions. A nonselective metal chelation may cause a depletion of fundamental metal ions, including those of essential metalloenzymes. Third, the chelator molecule must be able to immediately complex the metal ions that are present in excess in the brain to reduce aggregation of amyloid proteins in the brain. Fourth, a successful metal chelator establishes a low toxicity and minimal side effects.

The focus of this Review Article is to exhibit the neuropeptides that may serve as potential metal chelators. Section 2 briefly demonstrates various metal chelators that have been proposed and used in clinical studies. In addition, it describes the disadvantages and the fails of the currently used metal chelators. In Section 3, a brief overview on structural characterization and the role of neuropeptides are summarized. The qualifications of neuropeptides to successfully bind metal ions are detailed by experimental and computational studies in Section 4. The roles and the effects of neuropeptides as therapeutic agents in neurodegenerative diseases are elaborated in Section 5. Finally, future perspectives and future studies are discussed in Section 6.

## 2 Metal Chelators

### 2.1 Metal Chelator Agents of Small Compounds: Activity and Toxicity

Transition metal ions are essential nutrients and play a crucial role in various types of protein cofactors. The excess of these metal ions may be available for toxic reactivity. These redox-active metals may induce the formation of toxic hydroxyl radicals that oxidize proteins and consequently lead to cell death. The redox-active metals cause oxidative stress and protein misfolding in neurodegenerative diseases, such as AD and PD. To inhibit the redox-active metals, small molecule chelating agents were investigated as a promising strategy for treating neurodegenerative diseases. Herein, we provide a short list of small molecule chelating agents. Further detailed small compounds were extensively reported in the literature.^7^

**Figure 1:**
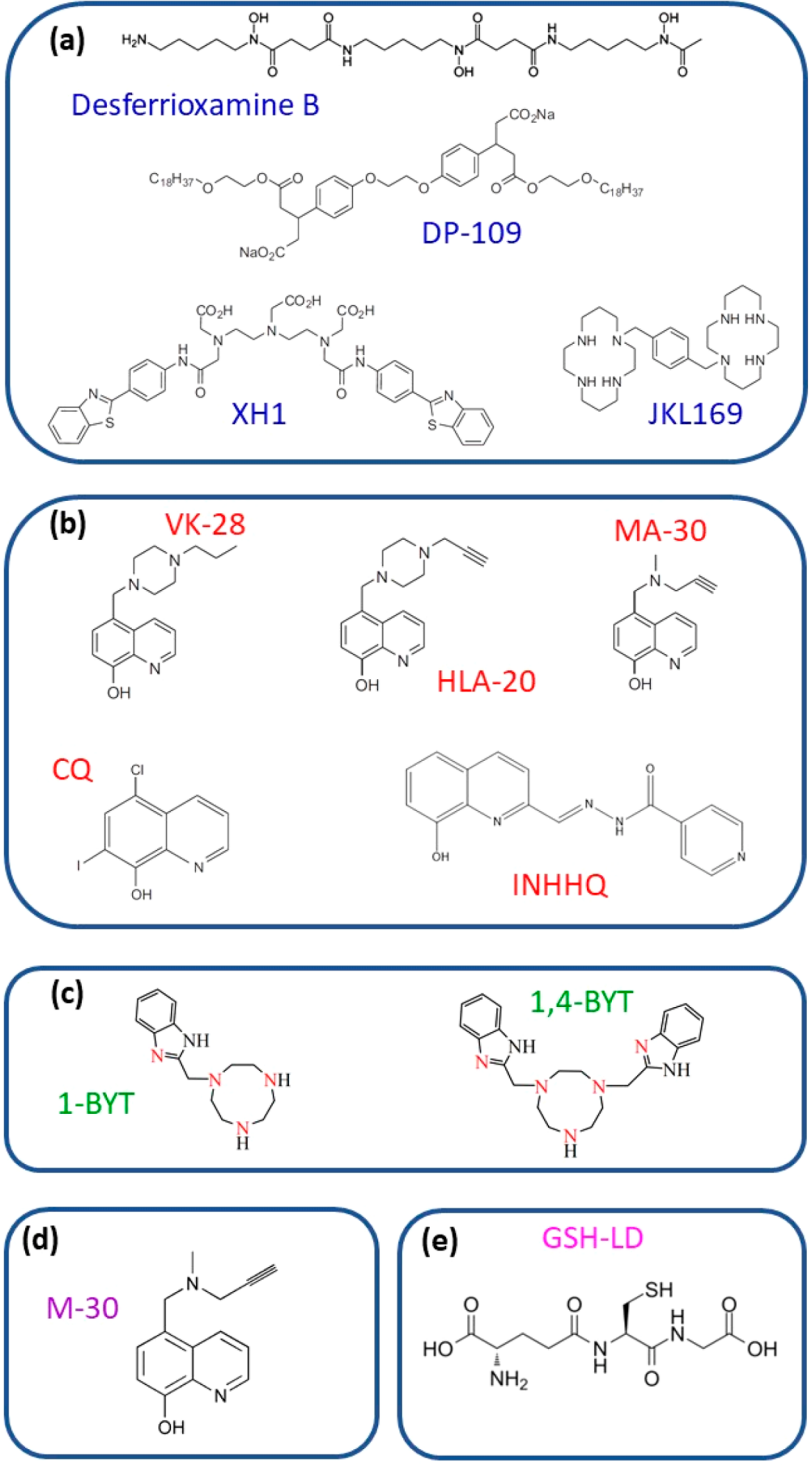
(a) First generation of synthetic small compound agents of metal chelators. (b) The metal chelators that are based on 8-hydroxyquinoline (8HQ). (c) Superoxide dismutase (SOD)-mimic compounds. (d) Metal chelator with monoamine oxidase (MAO) activity. (e) Peptide antioxidant ligand compound, glutathione.

The first compound that was used for metal chelation therapy in AD patients was for iron chelation: desferrioxamine B (Figure 1a).^8^ Later, other iron chelators were used, such as the lipophilic metal chelators DP-109 and DP-460 for AD and amyotrophic lateral sclerosis (ALS) mouse models.^9^ The use of the desferrioxamine B improved significantly the cognitive decline of AD patients, but this compound established several drawbacks: (i) the charged and the hydrophilic properties of the compound prevented BBB crossing, (ii) the compound was easily degraded, and (iii) due to the relatively high affinity of the metal to the compound, the patients suffered from side effects, such as anemia. The other lipophilic metal chelators were not used in clinical trials, probably due to the clinical outcomes of the desferrioxamine B treatments. Thus, these compounds were removed from the pharmacologic market.

The current Review Article focuses on Zn^2+^ and Cu^2+^ chelators since these metals are mostly common as cofactors that promote amyloid aggregation in neurodegenerative diseases. A further lipophilic metal chelator, XH1, is a compound that is based on a “pharmacophore conjugation” concept (Figure 1a). This molecule has a bifunctional activity: both amyloid binding affinity and Zn^2+^ chelating moieties. This was tested in mouse models.^10^ Derivatives of saturated tetraamine, such as the bicyclam analogue JKL169, exhibited decreased Cu^2+^ levels in the brain cortex in rats (Figure 1a).^11^ It was proposed that these compounds are capable of maintaining normal levels of Cu^2+^ in the blood, cerebrospinal fluid (CSF), and corpus callossum in rat, and thus, they may be candidates for AD treatments.^12^ However, these compounds were not investigated in clinical trials.

One of the strategies for developing chelating agents for neurodegenerative diseases is the “drug repositioning” or “drug repurposing”.^13^ A list of such compounds is reviewed elsewhere.^7^ The advantages of this strategy are the following: (i) there is a low investment in time and costs; (ii) the information on the pharmacokinetic, toxicology, and safety of these compounds already exists; and (iii) the compounds act via different mechanisms of action for AD treatments.^14^

The next generation of the development of metal chelation therapy was based on the 8-hydroxyquinoline (8HQ) analogues, such as VK-28, HLA-20, and MA-30 that were tested for iron chelation by an in vitro study (Figure 1b).^15^ One of the 8HQ analogues that was tested in a phase II clinical trial for AD and PD patients was the clioquinol (CQ) compound (Figure 1b).^16^ The CQ is part of the chelating agent class that is called “metal protein attenuating compounds” (MPACs). This class has comprehensive and important advantages in metal chelation therapy: (i) the compounds are capable of easily crossing the BBB; (ii) they are soluble and, thus, are able to decrease amyloid oligomerization and dissolve the amyloid plaques; (iii) they have subtle effects on metal homeostasis; and (iv) they have properties of rescinding the oxidation and the toxicity of Aβ peptide mediated by metal ions.

The second-generation compound of CQ (that is also known as PBT1) was a clioquinol-related compound, PBT2. This compound was shown to be a more promising drug than CQ. The PBT2 compound established a broad range of advantages:^17^ (i) it has a higher solubility than CQ, and thus, it easily dissolves Aβ oligomers and prevents the production of oligomers; (ii) it increases BBB permeability; (iii) it enhances cognitive functions in transgenic mice; (iv) it has fewer side effects compared with CQ; and (v) it has an easier chemical synthesis. However, even though PBT2 established good safety use and tolerability for patients with mild AD, the phase II clinical trial did not show a considerable drop in amyloid plaques.^18,19^

Additional 8HQ derivatives of metal chelators that were designed to interact with Cu^2+^ and Zn^2+^ with a high metal affinity include an arylhydrazone moiety, such as HPCIH and INHHQ (Figure 1b). The INHHQ compound (i) is capable of crossing the BBB, (ii) disrupts the interactions between Cu^2+^ ions and α-synuclein, and (iii) inhibits the oligomerization of the α-synuclein and, thus, may be a good candidate for PD treatment.^20^ The HPCIH compound illustrated a competition with Aβ for Zn^2+^.

Finally, superoxide dismutase (SOD)-mimic compounds were designed as metal chelating agents with an activity similar to that of CQ. Such SOD-mimic compounds include 1-BYT and 1,4-BYT (Figure 1c).^21^ The advantage of these compounds is the fact that the imidazole groups have several sites in which modifications can be performed in order to provide various properties for the compound with the aim of preventing Aβ aggregation. Therefore, these compounds may be a basic strategy for the development of novel candidates for treating neurodegenerative diseases.

Still, most of these small molecule metal chelating agents were tested in vitro and in vivo. Like various conventional therapies targeting amyloids, the outcomes of therapeutic metal chelation have shown disappointing results when translated to human clinical trials. Specifically, most of the large class of 8HQ derivatives did not progress to clinical trials, probably due to the failure of the CQ and PBT2 compounds.^22^ In summary, there has been a bias toward reporting outcomes of clinical trials of metal chelating therapeutics as positive and beneficial for patients. It may be that a new strategy of metal chelating therapeutics should be developed to treat neurodegenerative diseases.

### 2.2 Metal Chelators with Enzyme Activity

In addition to high levels of Fe^2+^ in the brains of AD and PD patients, there is also an increase in the activity of monoamine oxidases (MAOs), enzymes that oxidatively degrade neurotransmitters, such as dopamine, and consequently produce H_2_O_2_. These degraded neurotransmitters together with glutathione lead to oxidative stress.^15^ Therefore, iron chelation was used in clinical trials as a strategy to inhibit MAO activity.

Some of the iron chelating compounds that were designed to inhibit MAO include M-30 and HLA-20 (Figure 1d). These compounds are part of the derivative compounds of 8HQ. The HLA-20 compound has shown similar neuroprotective effects of rasagiline and selegiline, two MAO inhibitors that were used clinically for treating PD patients.^23^ In vitro studies illustrated that these bifunctional agents coordinate Fe^3+^ with ligand:metal complexes ratio of 3:1. These agents inhibit lipid peroxidation, establish modest MAO inhibitory activity, and demonstrate a reasonable cell permeability.^23,24^ These compounds show significant protective effects in cell culture models for studying neuronal oxidative stress and show a reduction of Aβ peptide levels.^24,25^

The iron chelation compound HLA-20 was rationally designed by incorporating the neuroprotective and neurorestorative propargyl-amine moiety from the anti-Parkinson drug rasageline into the VK28 compound. Similar to other nonspecific chelators, HLA-20 is capable of chelating metal ions both in the brain and in the body. To improve the permeability, improve the target specificity, minimize toxicity and side effects, and induce the efficacy of HLA-20 for treating AD patients, a leading strategy for a site-activated chelator acetylcholinesterase (AChE) inhibitor was designed. This designed metal chelator agent compound HLA-20A has a relatively low affinity for metal ions Fe^2+^, Cu^2+^, and Zn^2+^ but exhibits considerably lower cytotoxicity compared with that of HLA20. However, HLA20A can be activated following the inhibition of AChE with a concomitant release of active chelator HLA20.^26^ This led to the generation of a prochelator that requires AChE enzyme activation to release metal binding functionality.

### 2.3 Peptides as Metal Chelators

The MPACs class also comprises a peptidic ligand compound, such as GSH-LD (Figure 1e). GSH, known as glutathione, is an important antioxidant peptide that is composed of three amino acids, Cys, Gly, and Glu, and is presented in human cells. This peptide is known by its pleiotropic action in neurodegenerative diseases. l-Dopa (LD), also known as Levodopa and l-3,4-dihydroxyphenylalanine, is an amino acid that is produced in humans, and in some animals and plants. The synthesis of combined l-Dopa and GSH produces GSH-LD. This compound is capable of selectively removing the excess Cu^2+^, and partially removing the excess Zn^2+^, from Aβ peptides.^27^

Protein-derived bioactive peptides have been reported to trigger various physiological activities in the body and hence positively affect the health of humans. One of the activities is antioxidant mechanisms. Metal chelation is often studied as an indirect oxidant mechanism, because in metal complexation radical reactions in the chain are inhibited and oxidation phenomena are delayed. The radicals can react with biomolecules and consequently yield to disruptions and defects in biological tissues. The low concentrations of bioactive peptides in the body are one of the main factors that yield to the pathology cases in humans. Purified phaseolin and bean protein hydrolysates using pepsin and pancreatin have been assayed for antioxidant and metal chelating activities.^28^

Different amino acid residues may play a role in the antioxidant activity of peptides. The antioxidant activity usually depends on the chelation of the transition metal ions and the scavenging of free radicals. Nucleophilic sulfur-containing side chains in Cys and Met residues and aromatic side chains in Trp, Tyr, and Phe residues can easily donate hydrogen atoms. Thus, these residues are usually considered to have a potential antioxidant activity, although they may also have pro-oxidant effects under certain conditions.^29^ In addition to being susceptible to oxidative reactions, the imidazole group in His has metal chelating activity. Acidic and basic amino acids may also play a crucial role in Fe^2+^ and Cu^2+^ chelation.^30^ These peptides are related to food health and not related to neurodegenerative diseases.

Metal chelation of peptides in the chemistry of poly-His peptides was extensively studied in our group.^31−34^ In addition, extensive studies of metal chelation of peptides that are related to neurodegenerative diseases were reported elsewhere in experimental studies.^35,36^ The toxicity and the BBB permeability of these peptides were not investigated in the context of clinical trials for neurodegenerative diseases. A further group of peptides that can bind transition metal ions is neuropeptides. So far, there is very little information in the literature about metal chelation by neuropeptides. Section 3 introduces the features and the activity of neuropeptides in the human body, and their involvement in neurodegenerative diseases. Section 4 is focused on neuropeptides that potentially can be successful metal chelators. Examples of neuropeptides are presented from both experimental and computational studies. Finally, Section 5 discusses the potential of neuropeptides to serve as metal chelators.

## 3 Neuropeptides: Characterization and Functions

### 3.1 What Are Neuropeptides?

Neuropeptides are small molecules that are produced and released by neurons. The neuropeptides are part of a large class of signaling molecules in the nervous system, and they are considered to be key mediators in the communication between neurons and responsible for various brain activities.^37,38^ In addition to their activities in the brain, they expand in other tissues by the bloodstream and are involved in numerous mechanisms in the human body.^39^ Neuropeptides are involved in the secretion of salivary fluids, gastric fluids, intestinal fluids, and electrolytes. The action of neuropeptides is dependent on their binding to G protein-coupled receptors (GPCRs). The GPCRs can bind neuropeptides and thus may operate as useful peptides for therapeutics. Neuropeptides also function as cotransmitters of enteric cholinergic neurons, increase enteric neuron excitability, and consequently induce the release of enteric neurotransmitters, including acetylcholine.^40^ Neuropeptides are increasingly recognized as powerful modulators of the immune response, which is indicated by the fact that several immune cells produce neuropeptides.^41^ Neuropeptides have been implicated in the physiology and pathophysiology of chronic inflammatory diseases, such as asthma, allergic rhinitis, and chronic obstructive pulmonary disease. Extensive reported studies have provided information on the control role of neuropeptides in the normal respiratory functions, and in the involvement of regulatory neuropeptides in lung diseases.^42^

**Figure 2:**
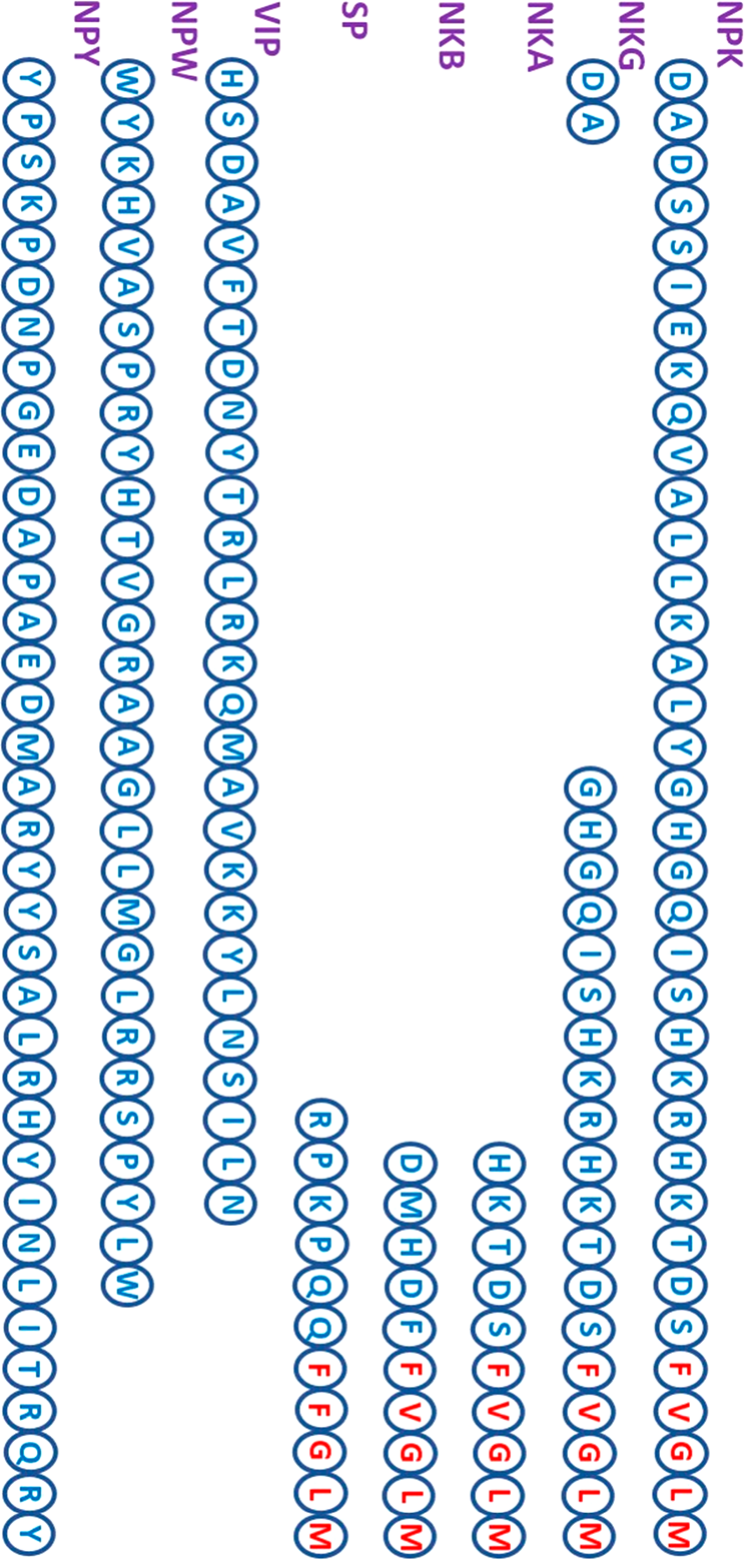
Sequences of neuropeptides. The neuropeptides NPK, NKG, NKA, NKB, and SP are part of the subfamily of tachykinins. The tachykinins consist of the conserved C-terminal sequence F-X-G-L-M, where X = F, Y, V, I. Some of the tachykinins have high similarity and identity along the sequence. NPW and NPY are rich in Trp and Tyr amino acids, respectively.

Neuropeptides comprise numerous subfamilies of groups, e.g., hypothalamic and other hormones, tachykinin, opioid peptides, and pancreatic polypeptides.^43−45^ To date, the most familiar peptides that were found and investigated include the following: neurokinin A (NKA), neurokinin B (NKB), substance P (SP), neuropeptide K (NPK) and neuropeptide gamma (NPG) (Figure 2). These five neuropeptides are part of the subfamily of tachykinins. A neuropeptide that is part of the hypothalamic hormones is named “oxytocin”.

The wide range of distribution of the neuropeptides in the signaling system in the human body is varied and extensively reviewed elsewhere.^46^ Some of the neuropeptides are distributed in the central nervous system (CNS); others are in the peripheral nervous system (PNS), and others are in the peripheral tissues.^46^ Herein, we provide a few examples of the distribution of only several neuropeptides. One of the neuropeptides that is largely expressed in the CNS is NKB, while other neuropeptides such as NKA and NPG are commonly present in the PNS.^47^

The neuropeptide SP for instance is expressed both in the PNS and the CNS. The neuropeptide W (NPW) has been reported to be present in a wide range of peripheral tissues, such as the heart, aorta, esophagus, stomach, small and large intestine, liver, spleen, lymph nodes, thymus, muscle, fat, lung, trachea, kidney, bladder, pancreas, adrenal gland, thyroid gland, submandibular gland, parotid gland, uterus, ovary, testis, epididymis.^48^ The NPW consists of Trp residues located at N-terminal and C-terminal of the peptide (Figure 2). This neuropeptide is not a part of the subfamily of tachykinins. Finally, the neuropeptide that is also not part of the subfamily of tachykinins NPY consists of multiple Tyr residues along the sequence (Figure 2); thus, it is named “NPY”.

### 3.2 Structural Characterization and Recognition Motifs of Neuropeptides

Neuropeptides are typically 3–100 amino acid residues long. The largest groups of the neuropeptides are characterized by their relatively short sequence: 3–40 amino acid residues. Most of the neuropeptides contain 3–20 amino acid residues, while others comprise 21–40 amino acid residues. Table 1 exhibits a partial list of neuropeptides. Principally, the amino acid composition of neuropeptides demonstrates that the residues Leu, Ala, Ser, Glu, and Gly are more abundant while the residues Trp, Cys, Met, His, and Tyr are the least abundant (Figure 2).

**Table 1:** Neuropeptides, Their Length Sequences, Their Structural Features, and Their Neuroprotective Effects

neuropeptide	no. of amino acids	structural features	PDB ID code	some neuroprotective effects
NPK	36	α-helix	2B19	induces concentration-dependent relaxations of precontracted cerebral arteries
NPG	21	α-helix	2MCE	acts on the hypothalamic pituitary gonadal axis to regulate functions related to reproduction
NKA	10	α-helix	1N6T	associates with the inflammatory cytokines
				protects neuronal cells from an excitotoxic insult
NKB	10	α-helix	1P9F	protects against copper-induced calcium channel opening and the synaptic homeostasis
SP	11	α-helix	2KS9	prevents cognitive impairments
				reverses cell death
				reverses K^+^-induced apoptotic cell death and amyloidogenic processing of APP
				reduces Aβ plaque deposition in the cortex
VIP	28	α-helix	2RRI	rescues impaired recognition
				stimulates the nonamyloidogenic processing of APP
				reduces Aβ40 and Aβ42
				rescues Aβ-induced cell death
NPW	30		a	plays role as physiologically relevant messenger in the brain networks and activates hypothalamic pituitary adrenal
NPY	36	α-helix	1RON	prevents depressive-like behavior and spatial memory deficits
				attenuates ER stress-induced cell death
				rescues Aβ-induced cell death
				ameliorates neurodegenerative pathology
				protects human neuronal cultures

a Not available in the Protein Data Bank.

**Figure 3:**
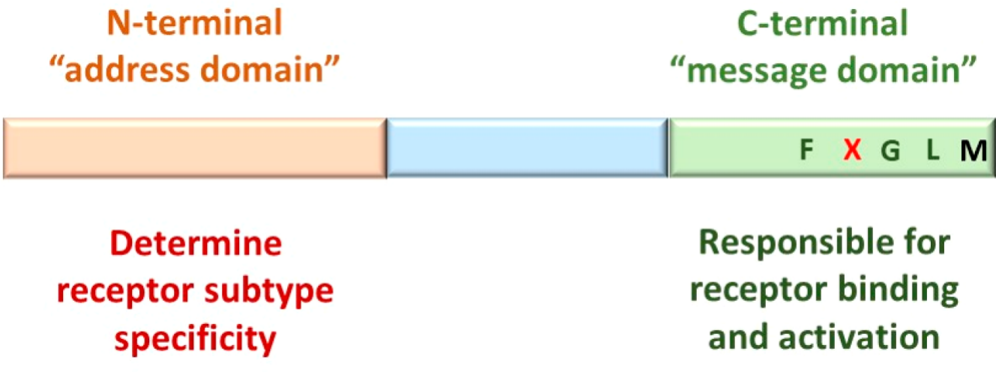
Characterized domains in tachykinins along the sequence. The N-terminal domain is named the “address domain” and determines the receptor subtype specificity. The C-terminal domain is named the “message domain” and is responsible for receptor binding and activation.

There are at least thousands of neuropeptides that are divided into hundreds of families. This Review Article does not provide detailed information for all the hundreds of families but instead focuses on the most common family that is named “tachykinin”. The tachykinin family of neuropeptides consists of the conserved C-terminal sequence Phe-X-Gly-Leu-Met-NH_2_, where X represents either an aromatic (Phe or Tyr) or a branched hydrophobic (Val or Ile) residue.^39,49^ In addition, the tachykinin family comprises an amidated C terminus, which is crucial for the biological activity of the neuropeptides. The synthesized deamidated tachykinins demonstrated an inactivity.^50^ Experimental studies revealed that the amidated C-terminus and a minimum chain length of six amino acid residues are crucial for peptide activity.^51^ The N-terminal domain of the tachykinin family is named the “address domain”, and it differs in the lengths and the types of the amino acid sequences among the various neuropeptides (Figure 3). It was hypothesized that this address domain plays a crucial role in determining the receptor subtype specificity. The C-terminal domain of the tachykinin family is named the “message domain”. This domain is responsible for receptor binding and activation (Figure 3).^16,52^

The structural features of tachykinins are dependent on different environments and have been widely investigated. It has been reported that tachykinins demonstrate some properties of secondary structures (i.e., β-strands and/or α-helices) in a solution environment, but these tachykinins also undergo a rapid conformational change, thus yielding to an ensemble of conformations.^53^ Recently, we have shown a conformational change in solution for some of the tachykinins applying experimental and computational tools.^54^ Interestingly, because of the rapid conformational change, the experimental techniques cannot be capable of recognizing all of the conformations of a particular tachykinin. For instance, a circular dichroism (CD) measurement of NKA in a solution environment showed a random coil structure by CD.^55^ However, several partially helical structures of NKA in solution were distinguished using molecular dynamics (MD) simulations.^54^ The explanation of the disagreement is due to the rapid conformational change that is seen in the MD simulations, and the experimental techniques are limited to introduce this process. Finally, in the presence of the membrane model, such as dodecylphosphocholine (DPC) micelles, the tachykinins exhibit helical conformations due to the hydrophobic environment.^55^

### 3.3 Role of Neuropeptides in Human Body

Neuropeptides are the most diverse class of signaling molecules engaged in numerous biological and physiological functions in the brain and in other peripheral organs. In addition to these numerous functions, neuropeptides act also as neurotransmitters and neuromodulators.^56^ Specifically, the neuropeptides are released from the synapses and mediate the communication between the neurons and effector cells. Neuropeptides and their receptors play an important role in several key processes. When neurons release neuropeptides, the binding of the neuropeptides to their receptors yields to conformational changes within the receptor that may either lead to open ion channels or activate coupled G proteins that consequently can cause a series of downstream effects within the cell.^57^

The tachykinin family has a wide range of physiological activities that have been attributed to the lack of specificity of the tachykinins for a particular receptor type.^58^ The lack of the specificity for a particular receptor type is probably due to the conformational flexibility and the short-length sequence of the tachykinins.^58^ The tachykinins complete their activity through membrane bound receptors that are part of the G-protein receptors and that share a high sequence similarity. Three pharmacologically distinct receptor subtypes that have been identified for tachykinin are NK-1, NK-2, and NK-3.^58^ All tachykinins bind to all of the receptor subtypes. However, some of the tachykinins prefer to bind only to one receptor even though there is lower affinity. Examples follow: substance P (SP) prefers to bind to NK-1, NKA selects NK-2 for binding, and NKB adopts binding to NK-3.^59^ Therefore, the tachykinins have the ability to disperse in abundant types of tissues in the human body.

Furthermore, neuropeptides have been implicated in the regulation of normal biological functions, e.g., feeding regulation,^60,61^ adaptation to external factors, such as temperature fluctuation,^62^ and to internal stress factors, including depression, anxiety, and post-traumatic stress disorder.^63^ Several of the prior isolated mammalian neuropeptides are hormones that are released from the gut, the adrenal glands, and the pituitary.^64^ For instance, the gut is one of the most abundant sources of SP, which is synthesized by enteric cholinergic motor neurons, and localized in CNS and PNS, as well as in the gastrointestinal tract, colon, and intestine.^65^ Additional hormones that regulate diuresis and lactation and influence social behavior include the hypothalamic neuropeptides vasopressin and oxytocin.^66^

Extensive studies have provided evidence regarding the roles of neuropeptides for regulation of neuronal biological functions such as learning and memory,^67^ cognition and emotion,^68^ and body temperature and stimulation of food intake.^69^ The involvement of neuropeptides in the aging processes is well-known. For instance, it has been shown that NPY is capable of interfering with several aging hallmarks, such as loss of proteostasis and stem cell exhaustion, and altering intercellular communication.^70^ Moreover, an in vivo study exhibited that transgenic mice models at high levels of NPY live longer than in the absence of or presence of a low level of NPY.^71^ It was also shown that neuropeptides have some effects on the cardiovascular system, and particularly on blood pressure. However, these effects depend on the concentration dose. For instance, at low doses of NPY, there are protective effects, while at high doses there are pathogenic effects.^72^ Finally, NKA and its N-terminal extended form, NPG (Figure 2), are involved in other processes, such as regulation of endocrine, stimulation of salivary secretion, bronchoconstriction, and vasodepression.^73^ There are other functions in which neuropeptides are involved in the brain and other peripheral tissues that are detailed and reviewed elsewhere.^74^ The current Review Article is focused on the metal chelation role of the neuropeptide; thus, herein, we provided only a brief description of some of the functions and activities of a few neuropeptides.

### 3.4 Involvement of Neuropeptides in Neurodegenerative Diseases

Neuropeptides comprise numerous subfamilies of groups. One of the common subfamilies of these groups that is involved in neurodegenerative diseases is the tachykinins. The tachykinins are broadly known and recognized by their involvement in neurogenerative disorders, such as AD, PD, Huntington’s disease (HD), Machado–Joseph disease (MJD), schizophrenia, and epilepsy.^63^ Some of the neuropeptides are densely localized in cognition-related brain regions, and their involvement in such neurodegenerative diseases and in their pathophysiological mechanisms is well-established. Herein, we briefly review 10 neuropeptides that are very common and known to relate to AD, PD, HD, and MJD.

#### 3.4.1 Involvement of NPY in AD, PD, HD, and MJD

The involvement of NPY in AD, PD, HD, and MJD has been extensively reviewed elsewhere.^75^ The high concentrations of NPY in the CNS are mostly present in the hippocampus, cerebral cortex, thalamus, brain stem, and cerebellum. The biological functions of NPY are activated by binding to various NPY receptors in several brain regions. For instance, NPY affects cell migration, cytokine release, and antibody production through its Y1 receptors.^76^ Neuronal loss is one of the hallmarks of the pathological features of AD. It is well-known that NPY has neuroprotective effects in AD.^77,78^ Neuronal replacement therapies have been reported for the treatment of neurodegenerative diseases.^79^ Neuropeptides such as NPY can dynamically regulate adult neurogenesis.^80^ A lentiviral vector expressing NPY, which was fused to a brain transport peptide (apolipoprotein B) for widespread CNS delivery in an amyloid precursor protein of an AD mouse model, was developed in order to explore the role of NPY in neurogenesis of AD.^78^ The results showed that the proliferation of neural precursor cells in the subgranular zone of the hippocampus increased significantly without further differentiation into neurons. Moreover, pretreatment with NPY protects neurons against Aβ neurotoxicity which is accompanied by an increased intracellular level of nerve growth factor (NGF)^81^ and brain-derived neurotrophic factor (BDNF).^82^ Immune response also plays an important role in the pathogenesis of AD. It was reported that NPY can suppress neuroinflammatory responses and neurodegeneration by delivering NPY-apolipoprotein B to the brain, and consequently lead to a widespread reduction in astrogliosis. Therefore, NPY affects the attenuation of neuroinflammation by activating the two receptors of NPY: Y1 and Y2.^78^

NPY is also involved in pathogenesis of PD. It has been shown that the loss of the nigrostriatal dopamine pathway led to a significant increase in the number of NPY-expressing cells in the striatum in animal.^83^ Moreover, NPY demonstrated interactions between glutamate and dopamine-containing neurons.^84^ It has been illustrated that the NPY protects dopamine neurons by inhibiting the release of glutamate in PD. Finally, it has been reported that striatal cells are the targets of cortical glutamatergic neurons.^85^

Huntington’s disease is an autosomal dominant inherited degenerative disease of the nervous system, specifically the lesions in the cerebral cortex and striatum. The pathological process of HD can lead to encephalatrophy, especially causing damage in the striatum.^86^ NPY is expressed by medium-sized γ-aminobutyric acid (GABA) ergic neurons in the striatum, which receives inputs from both cortical glutamatergic and nigral dopaminergic neurons and connects with neighboring cells. It has been reported that the expression of NPY in HD was increased in the basal ganglia, the cortex, and the subventricular zone.^87^ The activation of NPY as a potential therapeutic target in the mice model of HD was investigated.^88^ This in vivo study showed an increase in survival time and ameliorated the associated motoric and cognitive symptoms with NPY treatment. Finally, it was shown that NPY is also involved in inhibiting glutamate release, which consequently may reduce glutamate excitotoxicity in HD.^88^

MJD is a rare and progressive autosomal dominant neurodegenerative disorder and has features similar to those of other polyglutamine diseases such as HD.^89^ An in vivo study illustrated that NPY overexpression alleviated motor coordination and balance disabilities, prevented an increase of the mutant ataxin-3 induced in microglial immune reactivity, up-regulated BDNF levels, and reduced the proinflammatory cytokine IL-6 mRNA levels in an MJD mouse.^90^ The increasing levels of BDNF and reduction of neuroinflammation demonstrated the beneficial effects of NPY on MJD. Finally, it has been reported that NPY also plays a crucial role in increasing the levels of serotonin and norepinephrine, decreasing hypothalamic pituitary adrenal (HPA) axis hyperactivity, and reducing the plasma adrenocorticotropic hormone and cortisol plasma levels.^91^

#### 3.4.2 Involvement of Tachykinins in AD, PD, and HD

The tachykinins exhibit complicated functions in the CNS, because these neuropeptides are expanded in various regions in the brain. The tachykinins protect against the neurotoxic processes of AD,^45^ PD,^92^ and HD.^93^ The tachykinin family includes a long list of peptides among them, and the most commonly studied peptides are NKA, NKB, and SP. In the current Review Article, we will focus on the involvement of these three tachykinins.

The SP plays a crucial role in memory modulation: it has been shown that SP improved learning and memory in animal models.^94^ Early studies revealed that the expression of SP is altered in different regions in the brain of AD patients. The levels of SP decrease in the cortex, hippocampus, and striatum in AD patients and animal models.^95^ Interestingly, it was reported that in patients with late onset AD (over 65 years) the levels of SP-immunoreactivity were significantly higher than in patients with early onset.^96^ In PD, a 30–40% decrease of SP concentrations was detected in the pallidum and substantia nigra, which is likely to be caused by an increased SP metabolism.^97^

It has been reported that intraseptal injection of both NKA and NKB increases the levels of acetylcholine in the hippocampus and amygdala, but not in the basal forebrain.^98^ It was found that NKB inhibits the neurotoxic effect of Aβ peptides in cultured neurons, acting as an antioxidant.^99^ NKB protects against copper-induced Ca^2+^ channel opening^47^ and synaptic homeostasis.^100^ NKA has also been associated with the inflammatory cytokines IL-1 and IL-6.^101^ Finally, it has been shown that levels of NKA are reduced in HD.^93^ It has been shown that NKA and NKB were more capable of protecting the neuronal cells from an excitotoxic (kainic acid) insult.^102^ Interestingly, the tachykinins share a homology sequence with an Aβ_25–35_ fragment.^99^ The homology sequences with the Aβ_25–35_ fragment allow these tachykinins to coaggregate with the Aβ_25–35_ fragment peptide to form amyloid fibrils; however, these aggregates decrease the toxicity.^103^

#### 3.4.3 Involvement of Vasoactive Intestinal Polypeptide (VIP) in AD and PD

The neuropeptide VIP is also named “pituitary adenylate-cyclase activating polypeptide” (PACAP) (Figure 2). The VIP is known as a neurotransmitter, neuromodulator, neurotrophic agent, and neuroprotective agent. It is widely spread in the peripheral and in the CNS.^104^ It has been reported that VIP is associated with the pathology of AD.^105^ It was shown that the levels of the VIP are reduced in several areas in the brain of AD patients.^106^ It was revealed that the low levels of VIP are due to tau and Aβ plaques, that consequently decreased recognition memory with aging.^107^ Moreover, it has been demonstrated for the first time ever that VIP protects against Aβ-induced neuronal toxicity.^108^ Furthermore, it has been exhibited that VIP increases the level of Aβ-degrading enzyme and, consequently, inhibits Aβ aggregation.^109^ It is thus suggested that an intranasal spray of VIP increases the levels of brain-derived neurotrophic factor (BDNF) and antiapoptotic Bcl-2 protein and, consequently, is a useful treatment for AD patients.

VIP also participates in the pathophysiology of PD.^110^ It has crucial activities against PD: not only is it neurotrophic, antiapoptotic, anti-inflammatory, and antioxidant, but also it counteracts motor deficits.^111^ The VIP exhibited neuroprotective activity in a rat model of PD.^112^ Therefore, it is proposed that VIP may also be useful for therapeutics for PD patients.

#### 3.4.4 Involvement of Orexin, Galanin, and Somatostatin/Cortistatin in AD and PD

Orexin is a hypothalamic neuropeptide that plays a crucial role in maintaining wakefulness. It is well-known that one of the clinical symptoms of neurodegenerative diseases is a sleep disturbance. Thus, the involvement of the orexinergic system in the pathophysiology sleep disturbances in AD patients was extensively investigated.^113^ In addition, it was shown that orexin inhibits Aβ uptake and induces degradation in microglial cells.^107^ Orexin has also exhibited neuroprotective activity in mice models.^114^

Galanin is widely distributed in the central and peripheral nervous system and the endocrine system. The amino acid sequence of galanin is highly conserved (almost 90% among species), which indicates the importance of this neuropeptide. Galanin plays crucial roles in memory and learning and has possible involvements in the therapeutics of neurodegenerative diseases.^115^ Interestingly, it has been shown that there is a functional link between galanin and cholinergic in AD.^116^ Moreover, production of amyloid plaques induces formation of hippocampal cholinergic and galaninergic neurons in a transgenic mouse model.^117^ This confirms the neuroprotective role of galanin against Aβ toxicity. Finally, an increase of galanin autoantibody levels has been exhibited in the cerebrospinal fluid of AD patients,^118^ probably due to the increase of Aβ toxicity.

Somatostatin is a cyclic neuropeptide and has many regulatory functions and inhibitory effects across multiple systems throughout the body. Specifically, in this Review Article we focus only on functions that are related to the neurology system. Somatostatin has been detected in several brain regions playing a role in learning processes, e.g., the amygdala, hippocampus, striatum, and cerebral cortex.^119^ Somatostatinergic systems in the brain are crucial in several physiological and pathological neuronal functions and in neurodegenerative diseases.^120^ Moreover, it was shown that somatostatin is involved in cognitive and emotional processes by modulating the function of the respective brain areas.^121^ Finally, lower levels of somatostatin in the cerebral cortex and cerebrospinal fluid are typical hallmarks of AD patients.^122^

## 4 Identification and Basic Elements of Neuropeptides to Bind Metal Ions

### 4.1 Basic Properties and Features of Neuropeptides

Metal ions are crucial for critical biological functions in human body. In some cases, the presence of metal ions is important for human health, and in other cases these metal ions are involved in diseases. In fact, metal ions can bind and orient a substrate with respect to functional groups in the active site of proteins. The ligands in proteins that could coordinate metal ions are known as side chain carboxylates, sulfur atoms, and imidazole groups. These groups are recognized in aspartic acid, glutamic acid, methionine, histidine, and cysteine.^123^ These amino acids appear in the sequences of numerous neuropeptides, thus allowing them to bind metal ions. The coordination of histidine residues within neuropeptides to Cu^2+^ ions has been investigated for NPY^124^ and NKB.^47^

**Figure 4:**
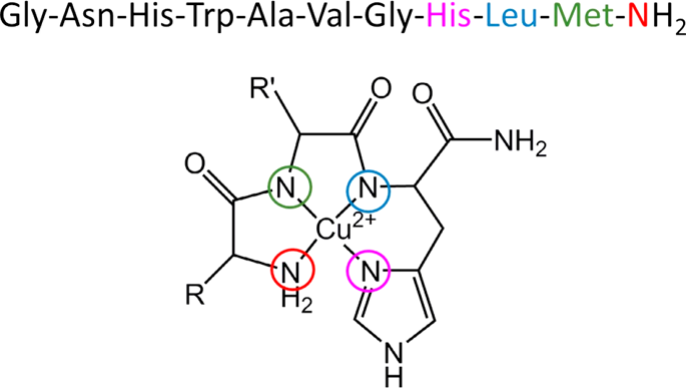
Amino terminal Cu and Ni (ATCUN) binding motif. The metal binds amino terminal nitrogen, histidine imidazole nitrogen, and histidine amide nitrogen at the third position. This metal binding site is found in the N-terminal of numerous natural proteins and peptides. The neuropeptide neuromedin C binds Cu^2+^ with N-terminal nitrogen (color: red), imidazole nitrogen of His8 (color: pink), and amide nitrogen atoms of Leu (color: blue) and Met (color: green).

The “amino terminal copper and nickel” (ATCUN) motif was first characterized in human serum albumin.^125^ This motif exhibits the binding of a copper ion by amino terminal nitrogen, histidine imidazole nitrogen, and histidine amide nitrogen at the third position (Figure 4). This motif has been recognized in numerous neuropeptides,^126^ e.g., NKB,^126^ neuromedin C,^127^ and more. Therefore, functionalizing neuropeptides with metal chelating groups is an important strategy to target agents to specific brain locations.^128^

### 4.2 Metal Binding Sites and Affinity in Neuropeptides: Experimental Output

Neuropeptides have the capability of binding metal ions, and the affinity for each metal ion may vary due to the type of metal, due to the type of neuropeptide, and obviously due to the experimental conditions. Metal coordination number (i.e, the ability to bind to a given number of ligands) and molecular geometry are shared properties between a ligand and its cognate metal. The metal coordination and the molecular geometry of the metal–ligand complex are proposed to be a key determinant of specificity. There are numerous neuropeptides that can potentially bind metal ions. However, to date, there are a limited number of experimental studies that investigated only several neuropeptides that bind metal ions. So far, the experimental studies investigated metal ions that bind to the tachykinins NKA, NKB, SP, NPG, and NPY. The following sections present the experimental studies that demonstrated the ability of these tachykinins to bind metal ions.

#### 4.2.1 Characterization of Metal Binding Sites in NKA

NKA is involved in various processes, such as regulation of endocrine, stimulation of salivary secretion, bronchoconstriction, and vasodepression.^73^ In fact, this neuropeptide was first purified in 1983 from a porcine spinal cord,^129^ and the first work that investigated its ability to bind Cu^2+^ ions was completed in 2010.^49^ This work characterizes the Cu^2+^ binding site in NKA and investigates the reaction of Cu^2+^ and NKA in the presence of hydrogen peroxide. The metal-catalyzed oxidation reaction produces reactive oxygen species (ROS) that are generated in neurodegenerative diseases.

Table 2 summarizes the specific metal binding sites within NKA at different pH values. In the 3.5–7.5 pH range, several monomeric Cu^2+^–NKA complexes were produced. The dominant Cu^2+^–NKA complex exhibited that the Cu^2+^ ion coordinates with amine and imidazole nitrogens of a His residue. In the 6.5–8.5 pH range, a distinct complex was observed: the imidazole nitrogen serves as a bridge between two NKA monomers. Thus, one Cu^2+^ ion binds the amine nitrogen of His1, the amide nitrogen of Lys2, and the hydroxy group of Ser5, and the fourth coordinate is occupied by the imidazole nitrogen of the adjacent NKA as a bridge. Additional to the characterization of the Cu^2+^-coordination mode with NKA, the effect of the hydrogen peroxide on the Cu^2+^–NKA complexes was investigated. The results of this reaction led to a reduction of Cu^2+^ to Cu^+^ and oxidation of histidine residue to 2-oxo-histidine in NKA. It has been hypothesized that both hydrogen peroxide species and Cu^2+^ ions are available in vivo and, therefore, yield to ROS formation. Hence, it is possible that this process is a primary contributor for aging, which constitutes a risk factor for neurodegenerative diseases, and NKA can be an excellent Cu^2+^ chelator to inhibit this process.

**Table 2:**
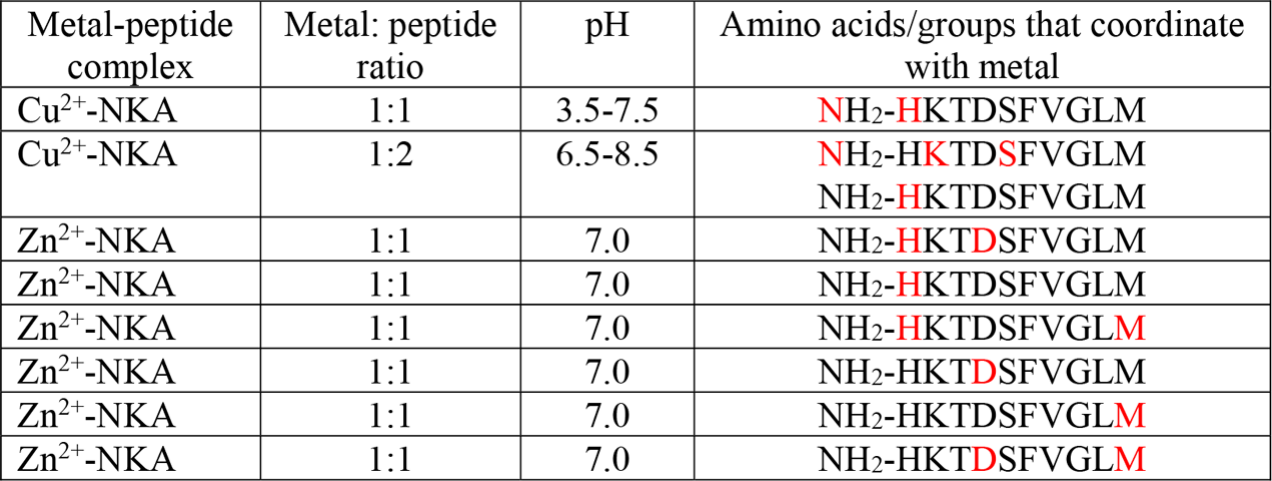
Amino Acid Residues and the Groups That Bind Metal Ions (Color: Red) in NKA with Diverse Metal:Peptide Ratios at Different pH Values^a^

a The atoms that bind the metal ions within each amino acid are nitrogen atoms. In some complexes, oxygen atoms within water molecules complete the coordination mode.

The Zn^2+^ binding site in NKA was also investigated by a combination of experimental and computational tools in our group.^54^ NKA has several residues that are capable of binding Zn^2+^ ions. The potentiometric titrations were applied to examine the specific Zn^2+^ binding site in NKA. It has been suggested that, at physiological pH, the Zn^2+^ ion binds mainly to the amine and imidazole nitrogens of His1. However, the molecular dynamics (MD) simulations for the Zn^2+^–NKA complex established seven distinct conformations with diverse Zn^2+^ binding sites. Six conformations of the Zn^2+^–NKA complex exhibited a random coil structure, and only one conformation of the complex presented a short helical structure. Interestingly, the Zn^2+^ ion was selectively transferred among the seven different Zn^2+^ binding sites, due to the absence of the diphenylalanine motif in the central domain of the peptide.^54^

#### 4.2.2 Characterization of Metal Binding Sites in NKB

Metal binding sites in NKB were relatively more investigated and reported compared to those of the other tachykinins. Herein, we present several experimental studies in which NKB can bind to several metal ions: Cu^2+^, Cu^+^, Ag^+^, Ni^2+^, and Zn^2+^. In some cases, the metal:NKB ratio in the complex was found to be 1:1, and in other cases, it exhibited a ratio of 1:2. Obviously, this and other structural features depend on the experimental conditions. Table 3 summarizes the specific binding sites of these metals.

**Table 3:**
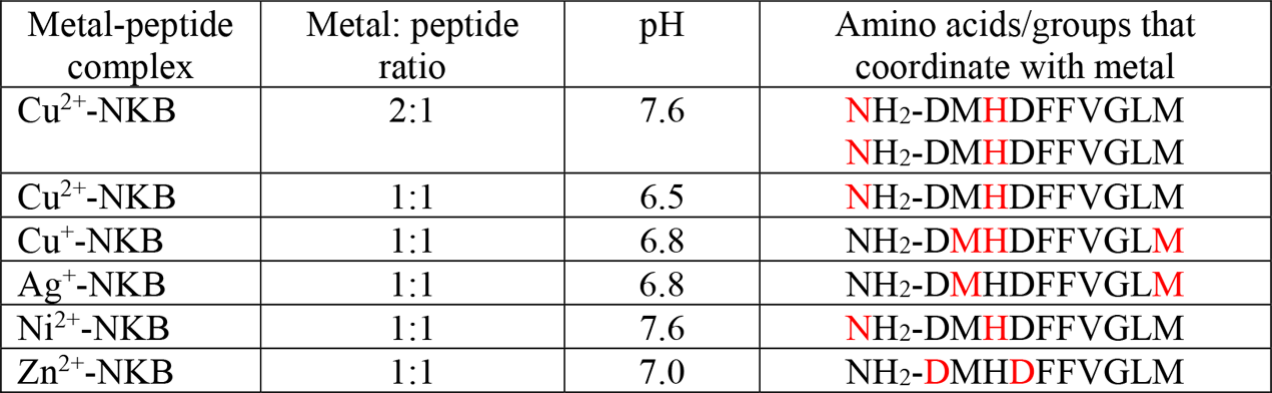
Amino Acid Residues and the Groups That Bind Metal Ions (Color: Red) in NKB with Diverse Metal:Peptide Ratios at Different pH Values^a^

a The atoms that bind the metal ions within each amino acid are nitrogen or sulfur atoms. In some complexes, oxygen atoms within water molecules complete the coordination mode.

The process of Cu^2+^ uptake into astrocytes is crucial and thus must be controlled. In case this process is not under control, the Cu^2+^ ions enter astrocytoma cells and cause the opening of the plasma membrane channels which consequently may lead to a damaging and dangerous disruption of the membrane.^130^ An experimental study illustrated that NKB can bind Cu^2+^. Importantly, the NKB limited the Cu^2+^ uptake level into astrocytoma cells and thus prevented the disruption of the membrane.^47^ These findings illustrate the crucial role of NKB in the regulation of synaptic Cu^2+^ homeostasis. Circular dichroism (CD) measurements demonstrated that one Cu^2+^ ion binds two NKB peptides, producing the [Cu^2+^(NKB)_2_] complex. NMR and EPR measurements have shown that each NKB peptide binds Cu^2+^ via a histidine imidazole and an N-terminal nitrogen, leading to a tetracoordinated binding mode. Finally, it has been illustrated that, in the absence of the Cu^2+^ ion, NKB exhibits a helical structure; in the presence of the Cu^2+^ ion, NKB is a random coil.^47^ It is important to note that a different experimental study illustrated that one Cu^2+^ ion binds one NKB peptide, producing the Cu^2+^–NKB complex.^131^

Interestingly, it has been found that NKB can also bind the Cu^+^ ion, which indicates the role of NKB in synaptic copper homeostasis and its involvement in redox cycling.^47,100^ Spectroscopic and electrochemical data demonstrated that NKB indeed binds Cu^+^ with an NKB:Cu^+^ ratio of 1:1. While the coordination geometry of the [Cu^2+^(NKB)_2_] complex is tetragonal, in the Cu^+^–NKB complex a quasitrigonal geometry was observed. The coordination involved Met2, Met10, and His at the third position, usually involved in Cu^+^ coordination. This coordination mode, specifically when Cu^+^ binds Met10, yields to a significant conformational change of NKB, which eventually loses its helical structure. Interestingly, it is known that the C-terminal Met10 in NKB binds to the receptors of NKB.^132^ Thus, it was suggested that the binding of Cu^+^ to Met10 may affect the activity of the receptors of NKB. However, to date, this assumption has not been approved. In summary, investigating the binding copper at different oxidative states is crucial and may provide insight into the role of NKB in protecting against AD.

It was also found that NKB can bind the Ag^+^ ion with a Ag^+^:NKB ratio of 1:1.^100^ The Ag^+^ coordination is different from the site adopted by Cu^+^. Spectroscopic data has shown that Ag^+^ does not bind to His3 in NKB, only to the side chain S atoms of Met2 and Met10. The Ag^+^ ion most likely forms a linear, two-coordinated complex with NKB with the two S atoms as ligands. Thus, it was proposed that, according to this coordination mode, while for Cu^+^ there is a conformational change of NKB, in Ag^+^ the NKB does not experience a large conformational change.

The His residue is located at the third position in the NKB peptide. This motif is known as an amino terminal copper and nickel (ATCUN) binding site. This ACTUN binding site is characterized by binding the Ni^2+^ ion with a Ni^2+^:protein ratio of 1:1.^131^ Thus, it is proposed that the geometry of Ni^2+^ in NKB is square-planar, producing four ligands with a Ni^2+^:NKB ratio of 1:1. However, this has not been approved either by experimental studies or by computational techniques, and future work should be initiated to examine this assumption.

So far, there is a lack of experimental studies with regard to Zn^2+^ ions that bind NKB. Recently, we investigated the binding site of Zn^2+^ ion in NKB peptide using computational tools.^54^ Our simulations demonstrated that the diphenyl alanine motif in the central domain of the peptide prevents hopping of the Zn^2+^ ion and, thus, conserves the Zn^2+^ binding site that involved O atoms of Asp1 and Asp4 and two water molecules.

#### 4.2.3 Characterization of Metal Binding Sites in NPG

**Figure 5:**
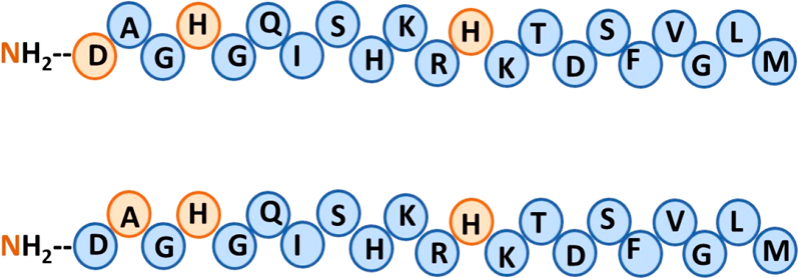
Two proposed Cu^2+^ binding sites (color: brown) in the neuropeptide NPG. The ratio of Cu^2+^:NPG in each one of the two Cu^2+^–NPG complexes is 1:1. The atoms that bind the metal ion are oxygen atoms, excluded for Ala. In Ala, the amide nitrogen binds the metal ion.

The effect of Cu^2+^-catalyzed oxidation on NPG has been investigated by experimental techniques.^133^ The potentiometric data at physiological pH proposed that there is an equilibrium between two Cu^2+^–NPG complexes (Figure 5). In the first complex, the Cu^2+^ ion binds to the N-terminal nitrogen, the β-carboxylate of Asp1, and the two imidazole nitrogens of His4 and His12 of NPG. In the second complex, Cu^2+^ ion binds to the N-terminal nitrogen, the protonated Ala2 amide nitrogen, and the two imidazole nitrogens of His4 and His12 of NPG.

As a successful demonstration of Cu^2+^ coordination with NPG, an oxidation reaction has been investigated in order to detect alternation in the metal binding site.^133^ In this reaction, hydrogen peroxide reduces Cu^2+^ to Cu^+^, and the Cu^+^ ion then was reacted with NPG. In this process, OH radicals were formed. The oxidation reaction thus led to a conversion of Met21 to methionine sulphone and to oxidation of His4 and His12 residues to produce 2-oxo-histidines. These types of reactions force a protein oxidation in which only a few specific amino acids are oxidized, particularly in the metal binding site domain, even though the oxidized metal complexes that were observed at physiological pH demonstrated similar coordinates as shown for Cu^2+^–NPG.

#### 4.2.4 Characterization of Metal Binding Sites in SP

**Figure 6:**
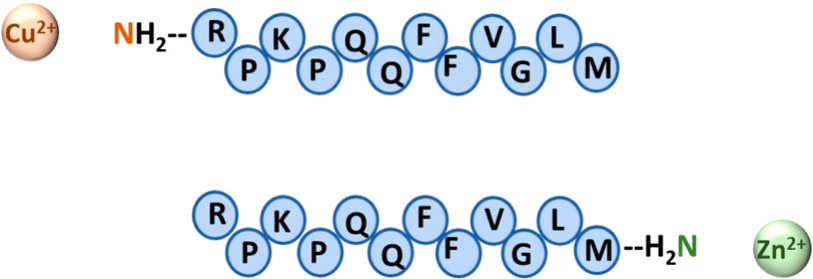
Cu^2+^ ion binds to the N-terminal nitrogen atom in the neuropeptide SP at pH > 10. At physiological pH (pH 7), the Zn^2+^ ion binds to the C-terminal nitrogen atom of the SP.

To examine whether SP is capable of limiting Cu^2+^ ion uptake into astrocytoma, an experiment in SDS solution that mimicked the lipid environment has been completed.^47^ It has been found that SP is not capable of binding Cu^2+^ ions at pH 7.6, and consequently, it does not inhibit uptake of Cu^2+^ ions into astrocytes. Interestingly, it has been shown that SP is not able to bind Cu^2+^ ions also in a solution environment at physiological pH.^47^ However, at basic pH (>pH 10), the N-terminal of the SP can bind Cu^2+^ ions (Figure 6).^134^

To date, experimental studies on Zn^2+^ binding sites in SP have not been reported. Recently, applying computational tools, we investigated the interactions of Zn^2+^ ion in SP peptide.^54^ Contrary to the numerous tachykinins, SP does not consist of residues prone to bind metal ions, such as His, Asp, Glu, etc. However, the carboxyl group of the C-terminal of SP has been proposed to be a potential site to bind Zn^2+^ ion. Hence, the Zn^2+^-SP complex was initially constructed while the Zn^2+^ ion was bound to the carboxylic group of the C-terminal domain. Molecular dynamics (MD) simulations confirmed that the Zn^2+^ coordination mode with two carboxylic oxygens atoms and two water molecules was conserved along all time scale of the simulations.^54^ Hence, it was proposed that Zn^2+^ ion binds to the nitrogen amide in the C-terminal od SP (Figure 6).

#### 4.2.5 Characterization of Metal Binding Sites in NPY

To date, the interactions between NPY and metal ions have been reported only for the Cu^2+^ ion. The interactions between Cu^2+^ ions and NPY have been investigated using extensive experimental techniques.^124^ It has been proposed that the Cu^2+^ ions may catalyze the production of reactive nitrogen species in the presence of nitrite. The complex Cu^2+^–NPY was formed under nitrative stress, which is expressed by the nitration of all five tyrosine residues (i.e., Tyr1, Tyr20, Tyr21, Tyr27, Tyr36) in the presence of NO_2_^–^, and consequently, this promotes the production of hydroxyl radicals. It was suggested that under these conditions the Cu^2+^ ion that binds the Tyr36 in the C-terminal of NPY to produce the complex may eliminate the interactions of the NYP with its receptors and, consequently, may prevent the biological activities of the NPY. However, these assumptions have not been approved. Finally, the UV–vis and ESI-MS spectra indicated that the NPY:Cu^2+^ ratio is 1:1, while one NPY peptide binds to one Cu^2+^ ion in aqueous solution. It has also been suggested that the His residue coordinates with Cu^2+^ via imidazole nitrogen; however, this assumption has not been confirmed.

**Figure 7:**
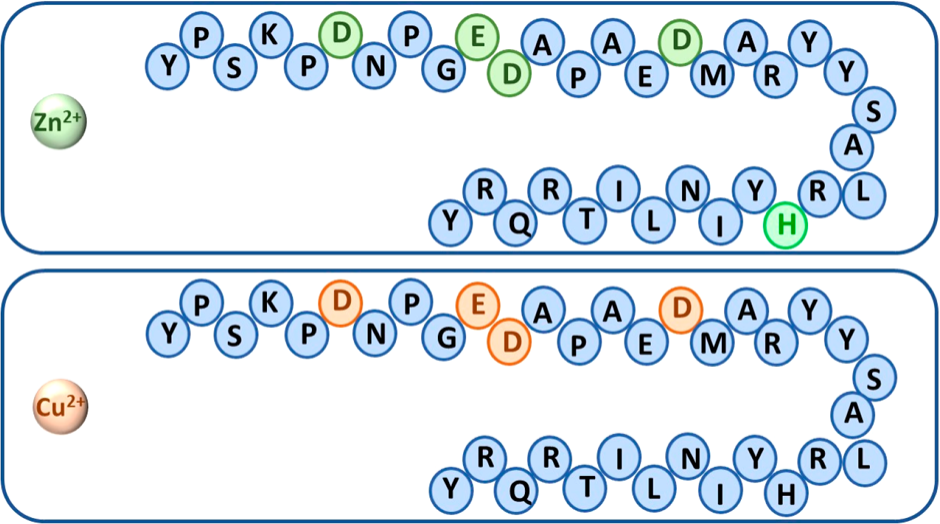
Zn^2+^ ions compete between two binding sites in NPY: two Zn^2+^–NPY complexes may be formed. The first Zn^2+^ binding site consists of Asp6, Glu10, Asp11, and Asp16, and the second Zn^2+^ binding site contains His26 and water molecules that complete the coordination mode. The NPY binds Cu^2+^ only in the N-terminal domain: Asp6, Glu10, Asp11, and Asp16. The Cu^2+^ does not bind the His26.

To date, experimental studies lack the specific metal binding sites in NPY. Recently, our simulations demonstrated that NPY could bind Zn^2+^ ion in two domains along the sequence of NPY (Figure 7). The first Zn^2+^ binding site consists of the residues Asp6, Glu10, Asp11, and Asp16, while the second Zn^2+^ binding site contains His26 and water molecules that complete the coordination mode.^135^ Our simulations also showed that NPY can bind Cu^2+^, but only along the N-terminal domain, containing the following residues: Asp6, Glu10, Asp11, and Asp16 (Figure 7). Our simulations, therefore, suggested that NPY can selectivity bind Cu^2+^ ions, while a competition between two binding sites may occur when NPY binds Zn^2+^ ions. The Cu^2+^ is not capable of binding His26. Several studies reported that imidazole and amino groups of His residues are involved in the chelate linkage between 1:2 molar ratio of Cu^2+^ and histidine.^136,137^

## 5 Neuropeptides as Potential Metal Chelators for Neurodegenerative Diseases

### 5.1 Role of Metal Ions in Amyloid Aggregation

The homeostasis of metal ions is essential for maintaining normal important biological functions in the brain. Variations in the balance of the metal ions in the brain are known to be related to formation of Aβ deposits and tau hyperphosphorylation or tau accumulation. Therefore, it was suggested that metal ions play a crucial role in the pathogenesis of AD. The imbalance of metal ions in the brain also initiates the formation of α-synuclein deposits that eventually lead to the pathogenesis of PD. The main metal ions that play a role in the pathology of AD and PD include Fe^2+^, Zn^2+^, and Cu^2+^. The specific roles of these metal ions in these neurodegenerative diseases have been extensively reported in the literature. Recently, the roles of these metal ions have been extensively reviewed for the pathogenesis of AD^138^ and PD.^139^

The common concept that has been stated in the scientific and medicinal literature is that these metal ions bind to the amyloid monomers and prompt the formation of toxic aggregates that lead to neuronal death. According to this concept, there have been proposals to design metal chelators to inhibit the process in which these amyloids will bind metal ions. The history of human clinical trials using compounds as targeting metals in the past four decades has been immensely reviewed.^140^ Currently, none of these designed compounds that target metal ions are used as a treatment for neurodegenerative diseases. The main reason that these compounds are not used as a treatment is due to their side effects. However, it is still considered that metal targeting compounds should be a promising treatment for neurodegenerative diseases.

### 5.2 Neuropeptides as Potential Therapeutic Metal Chelators for Inhibiting Amyloid Aggregation

In this Review Article, it was demonstrated in Section 3 that the neuropeptides have neuroprotective effects. Moreover, the neuropeptides are involved in numerous crucial biological functions in the brain. The ability of neuropeptides to bind metal ions was greatly detailed in Section 4. While there have been immense efforts to find optimal metal chelators, so far none of the designed metal chelators were found to be effective and safe. This Review Article proposes that neuropeptides may be ideal metal chelators to inhibit aggregation of amyloids in neurodegenerative diseases.

Neuropeptides comprise numerous subfamilies of groups, e.g., hypothalamic and other hormones, tachykinin, opioid peptides, and pancreatic polypeptides.^43^ Only a few neuropeptides were investigated as metal chelators by in vitro (reviewed elsewhere in Section 4) and by in silico studies.^54,135^ There are still large number of neuropeptides that need to be investigated for their ability to bind metal ions both by in vitro and in silico studies. Moreover, to date, in vivo studies have not been completed to investigate the neuropeptides as metal chelators in neurodegenerative diseases. Hence, it is necessary to complete future studies in mice models and in clinical trials on the effect of neuropeptides as metal chelators for treatment for AD and PD patients.

## 6 Summary and Future Perspectives

Neurodegenerative diseases are identified by protein amyloid aggregation in different regions in the brain. Metal dyshomeostasis is well-recognized as a crucial factor in neurodegenerative diseases.^1,2^ Metal ions, such as Cu^2+^ and Zn^2+^, are known to initiate amyloid aggregation and consequently yield the development of neurodegenerative diseases. Moreover, redox-active metals may induce the formation of toxic hydroxyl radicals that oxidize proteins and consequently lead to cell death. The redox-active metals cause oxidative stress and protein misfolding in neurodegenerative diseases. Therefore, there has been an extensive effort during the past four decades to develop metal chelators to inhibit amyloid aggregation.^140^ Most of the metal chelators are based on small chemical compounds. The disadvantages and the failings of the past and currently used metal chelators left behind crucial commitments to solve these barriers.

The research studies of some of the tachykinins as metal chelators have investigated the solution environment by experimental studies, and these studies were detailed here in Section 4. These studies demonstrated that these tachykinins are excellent metal chelators with high metal binding affinity. However, there are still, relatively, small numbers of experimental studies that examined the role of neuropeptides as metal chelators. Recently, a molecular modeling study demonstrated that three of the tachykinins indeed bind Cu^2+^ and Zn^2+^ ions.^54^ It is a hope that these studies will initiate future studies to investigate further neuropeptides that can bind metal ions. Importantly, the achievements from these studies may encourage investigations of these neuropeptides as metal chelators in vivo and later in clinical studies with AD and PD patients. Finally, future studies should be initiated to compare the neuropeptides’ metal chelation strength with that of small hydroxyquinoline based compounds, metal–amyloids, and metal–ACTUN peptide.
